# Maternal High-Fructose Corn Syrup Intake Impairs Corticosterone Clearance by Reducing Renal 11β-Hsd2 Activity via miR-27a-Mediated Mechanism in Rat Offspring

**DOI:** 10.3390/nu15092122

**Published:** 2023-04-28

**Authors:** Yuki Nouchi, Eiji Munetsuna, Hiroya Yamada, Mirai Yamazaki, Yoshitaka Ando, Genki Mizuno, Miyuki Ikeya, Itsuki Kageyama, Takuya Wakasugi, Atsushi Teshigawara, Yuji Hattori, Yoshiki Tsuboi, Hiroaki Ishikawa, Koji Suzuki, Koji Ohashi

**Affiliations:** 1 Department of Informative Clinical Medicine, Fujita Health University School of Medical Sciences, 1-98 Dengakugakubo, Kutsukake-cho, Toyoake 470-1192, Japan; 2 Department of Preventive Medical Sciences, Fujita Health University School of Medical Sciences, 1-98 Dengakugakubo, Kutsukake-cho, Toyoake 470-1192, Japan; 3 Department of Biochemistry, Fujita Health University School of Medicine, 1-98 Dengakugakubo, Kutsukake-cho, Toyoake 470-1192, Japan; 4 Department of Hygiene, Fujita Health University School of Medicine, 1-98 Dengakugakubo, Kutsukake-cho, Toyoake 470-1192, Japan; 5 Department of Medical Technology, Kagawa Prefectural University of Health Sciences, 281-1 Hara, Mure-cho, Takamatsu 761-0123, Japan; 6 Department of Medical Technology, Tokyo University of Technology School of Health Sciences, 5-23-22 Nishi-Kamata, Ota, Tokyo 144-8535, Japan

**Keywords:** DOHaD, epigenetics, pregnancy, glucocorticoid, 11 beta-hydroxysteroid dehydrogenase, fructose

## Abstract

We previously reported that maternal fructose consumption increases blood corticosterone levels in rat offspring. However, the underlying mechanism of action remains unclear. In the present study, we aimed to elucidate the molecular mechanism by which maternal high-fructose corn syrup (HFCS) intake increases circulating GC levels in rat offspring (GC; corticosterone in rodents and cortisol in humans). Female Sprague Dawley rats received HFCS solution during gestation and lactation. The male offspring were fed distilled water from weaning to 60 days of age. We investigated the activities of GC-metabolizing enzymes (11β-Hsd1 and 11β-Hsd2) in various tissues (i.e., liver, kidney, adrenal glands, muscle, and white adipose tissue) and epigenetic modification. 11β-Hsd2 activity decreased in the kidney of the HFCS-fed dams. Moreover, the epigenetic analysis suggested that miR-27a reduced *Hsd11b2* mRNA expression in the kidney of offspring. Maternal HFCS-induced elevation of circulating GC levels in offspring may be explained by a decrease in 11β-Hsd2 activity via renal miR-27a expression. The present study may allow us to determine one of the mechanisms of GC elevation in rat offspring that is often observed in the developmental origins of the health and disease (DOHaD) phenomenon.

## 1. Introduction

The developmental origins of health and disease (DOHaD) hypothesis suggests that early life nutrition and the environment have an impact on future health. Dutch famine cohorts have shown that individuals who experienced famine during pregnancy have an increased risk of obesity, dyslipidemia, and hypertension later in life [1,2]. Although starvation was originally thought to cause DOHaD, recent studies have shown that various nutrients also have an impact. Therefore, it is important to investigate the underlying mechanisms of DOHaD.

Fructose is a natural sugar that is found in fruits and vegetables. It has become increasingly popular in modern society owing to its high sweetness and low cost. It is commonly consumed in the form of high-fructose corn syrup (HFCS), which is present in many food and beverage products [3]. Epidemiological studies have suggested that increased fructose consumption is associated with metabolic dysfunction, including obesity, hypertension, dyslipidemia, and insulin resistance [4,5,6]. Our research has focused on the transgenerational toxicity of maternal fructose intake [7,8,9,10]. We have demonstrated that the offspring of rats fed with a 20% aqueous HFCS solution experience metabolic disorders, such as insulin resistance and hyperlipidemia, at 60 days of age [10]. Human studies have also demonstrated that the consumption of fructose-containing beverages during pregnancy is associated with metabolic disorders in children [11,12]. Therefore, maternal fructose intake induces DOHaD expression in both humans and rodents.

Glucocorticoids (GCs), namely cortisol in humans and corticosterone in rodents, are steroid hormones that are synthesized in the adrenal glands; they are involved in stress response [13]. Elevated circulating levels of GC cause insulin resistance, dyslipidemia, and hypertension [14]. GCs are metabolized by 11 beta-hydroxysteroid dehydrogenases 1 and 2 (11β-Hsd1 and 11β-Hsd2, encoded by *Hsd11b1* and *Hsd11b2*, respectively) [15]. 11β-Hsd1 regenerates active GCs, whereas 11β-Hsd2 converts GCs into their inactive forms. Studies have shown that maternal fructose consumption increases circulating GC levels in offspring [16,17], suggesting that maternal fructose-induced metabolic disorders may be caused by elevated GC levels. However, the underlying mechanisms are not fully understood.

Epigenetic modifications involve alterations in the DNA that affect gene expression without altering the underlying genetic code. These modifications are closely related to DOHaD and can be induced by environmental exposure during fetal development [18]. The most common types of epigenetic modifications are DNA methylation and the effects of microRNAs (miRNAs), which can either activate or repress gene expression [19]. We previously reported that excess maternal fructose causes epigenetic abnormalities in offspring [20,21], which may explain the increase in circulating GC levels.

In the present study, we aimed to investigate the molecular mechanisms underlying the increase in circulating GC levels in offspring of dams fed HFCS. We examined the activities of 11β-Hsd1 and 11β-Hsd2 in various tissues, i.e., the liver, kidney, adrenal glands, muscle, and white adipose tissue (WAT), of the offspring. We also investigated the epigenetic abnormalities related to changes in GC metabolism.

## 2. Materials and Methods

### 2.1. Animals

An experimental animal model was established, as described previously [10]. Briefly, Sprague Dawley (SD) rats (Japan SLC, Hamamatsu, Japan) were kept under standard conditions. After one week of acclimatization, the male and female rats were mated. After confirming pregnancy, the female rats were separated into two experimental groups: the first group was administered distilled water, while the second group was given a 20% aqueous solution of HFCS (Nihon Cornstarch Corporation, Tokyo, Japan) [10]. All animals were allowed ad libitum access to a certified standard laboratory rat diet (MF; Oriental Yeast, Tokyo, Japan). Distilled water and HFCS aqueous solutions were administered from the first day of pregnancy to postnatal day (PD) 21. Weaned male offspring were given standard chow and distilled water without restriction between PD 21 and PD 60. Weekly measurements of body weight and caloric intake in offspring were conducted during this period. After the experimental period, the offspring were subjected to a 6 h fast, followed by saline perfusion and isoflurane anesthesia. The serum, liver, kidney, adrenal glands, gastrocnemius muscle, and visceral white adipose tissue (WAT) were collected and stored at −80 °C until needed.

### 2.2. General Anesthesia

General Anesthesia was performed as previously reported [21]. Briefly, the anesthesia was induced and sustained with 4% isoflurane (Wako, Tokyo, Japan). The surgical level of anesthesia was determined by verifying the lack of response to a toe pinch in the animal’s respiratory pattern.

### 2.3. Quantification of Circulating Corticosterone Levels

The offspring sera were stored at −80 °C until required. Circulating corticosterone was measured with a MAGPIX-Luminex assay (Millipore) with a Rat Stress Hormone Magnetic Bead Panel.

### 2.4. Quantification of mRNA Expression

Total renal RNA was isolated using TRIzol Reagent (Thermo Fisher Scientific, Waltham, MA, USA) described in our previous report [22]. Quantification of mRNA was performed using a QuantStudio 7 Flex system (Thermo Fisher Scientific) and Thunderbird SYBR qPCR Mix (Toyobo, Osaka, Japan). The primer pairs used have been described previously [17]. To ensure accuracy in our results, we used *Actb* as an internal control to normalize the expression levels of the target genes. We then calculated the fold changes between the control and HFCS groups using the 2^−∆∆Ct^ method [23], which allowed us to accurately determine the changes in gene expression between the two groups.

### 2.5. Western Blotting

Kidney tissues were homogenized using radio-immunoprecipitation assay buffer (Wako Pure Chemicals, Osaka, Japan). Sodium dodecyl sulfate-polyacrylamide gel electrophoresis (SDS-PAGE) and Western blotting were performed as described previously [24]. The primary antibodies against 5α-Red1 (Abcam, Cambridge, UK), 11β-HSD2 (Proteintech, Rosemont, USA), and β-actin (Abcam, located in Cambridge, UK) were used. The immunoreactive bands were visualized using Immobilon Western Chemiluminescent HRP Substrate (Millipore, Billerica, MA, USA). The intensities of the specific chemiluminescent bands were analyzed using an ImageQuant LAS 3000 (GE Healthcare, Amersham, UK).

### 2.6. 11β-Hsd1 and 11β-Hsd2 Activity

We stored the liver, kidney, adrenal glands, gastrocnemius muscle, and WAT at −80 °C to avoid 11β-Hsd1 and 11β-Hsd2 activity. This method was based on that described in a previous report [25]. Briefly, the tissues were homogenized in 30 mM Tris buffer (pH = 7.4) containing 0.9 mM ethylenediaminetetraacetic acid and 0.3 mM sucrose to produce 40 mg/mL homogenates. The 11b-Hsd1 and 11b-Hsd2 activities were measured in the presence of cortisone and cortisol, respectively. Steroids were extracted with chloroform and 50 µM dexamethasone was added as an internal control. Reverse-phase HPLC analysis was performed on a 5C_18_-MS-II column (Nacalai Tesque Inc., Kyoto, Japan) at a column oven temperature of 30 °C and a detection wavelength of 254 nm. The isocratic mobile phase was methanol/water (60:40, *v*/*v*), and its flow rate was 0.6 mL/min. The analytes were identified using the following standards: cortisone (Merck, Darmstadt, Germany), cortisol (VWR International Ltd., Leuven, Belgium), and dexamethasone (Merck, Darmstadt, Germany). The HPLC measurement system used in the present study was linear in the range of 2–40 nmol for cortisol and cortisone levels, and all measured values were within this range. 11β-Hsd1 activity was calculated from the cortisol levels, and 11β-Hsd2 activity was calculated from the cortisone levels, which were adjusted to dexamethasone levels.

### 2.7. Analysis of Hsd11b2 DNA Methylation Levels in the Kidney

We determined the proportion of methylated CpG sites using bisulfite pyrosequencing, as previously described [21]. We selected the PCR primers using PyroMark Assay Design software version 2.0 (Qiagen, Hilden, Germany). The primer sequences were the same as those described previously [17].

### 2.8. Quantification of miRNA Expression

To synthesize cDNA from miRNAs, a miScript II RT Kit (Qiagen, Venlo, Netherlands) was used [26]. The concentration of miRNAs was determined using a Qubit miRNA Assay Kit (Thermo Fisher Scientific, Waltham, MA, USA). Quantitative polymerase chain reaction (qPCR) was performed using a miScript SYBR Green PCR Kit with miScript Primer Assays Rn_miR-27a_1, Rn_miR-103_2, Rn_miR-107_2, Rn_miR-422b_1, Rn_miR-194*_1, Rn_miR-92a-1*_1, and Hs_RNU6-2_11. We selected the miRNAs predicted to be biological targets of *Hsd11b2* mRNA with a context++ score percentile of 90% or higher using TargetScan, a web server that predicts biological targets of miRNAs. Among the selected miRNAs, we also selected those that were highly expressed in the kidney. For this purpose, we used the dataset reported by Kwekel et al. [27]. The PCR conditions were set as follows: 95 °C for 15 min, 40 cycles of 95 °C for 15 s, 55 °C for 30 s, and 72 °C for 30 s. We calculated the fold changes between the control and HFCS groups using the 2^−∆∆Ct^ method [26,28].

### 2.9. Transfection with miRNA Mimic

The rat epithelial cell line C9 was purchased from American Type Culture Collection (Manassas, VA, USA). C9 cells were cultured in advanced Dulbecco’s modified Eagle’s medium (Thermo Fisher Scientific, Waltham, MA, USA) supplemented with 10% fetal bovine serum, and seeded (7 × 10^3^) on 96-well plates. After 24 h, the C9 cells were transfected with the miScript miRNA mimic (Qiagen; the final concentrations of miR-27a and miR-107 were 0.1 nM and 10 nM, respectively) using Lipofectamine 3000 reagent. Negative control cells were transfected with small RNAs and scrambled sequences. The medium was changed 24 h after transfection, and after another 24 h, the cells were collected for the TRIzol reagent.

### 2.10. Luciferase Assay

The 3′ untranslated region of *Hsd11b2* mRNA containing the predicted binding sites was amplified by PCR using the following primers: miR-27a region, 5′-GCTCGCTAGCCTCGAGCCATTATAGACCCCTCA-3′ (forward) and 5’-CGACTCTAGACTCGAGCCACATCTCACGCTAAA-3′ (reverse); and miR-107 region, 5′-GCTCGCTAGCCTCGACTCTGGACCTCTCCTCTG-3′ (forward) and 5′-CGACTCTAGACTCGAAGCCAGTCAAGAGGGATT-3′ (reverse). The PCR amplicons were prepared using an In-Fusion HD cloning kit (Takara, Shiga, Japan) and inserted into the pmiRGLO dual-luciferase miRNA Target Expression Vector (Promega, Madison, WI, USA). The PCR products were inserted into the pmiRGLO dual-luciferase miRNA Target Expression Vector from Promega (Madison, WI, USA) with an In-Fusion HD cloning kit from Takara (Shiga, Japan). Subsequently, the vectors were transfected into the human hepatic cell line HepG II through the use of a Viafect Transfection Reagent from Promega (Madison, WI, USA). HepG II cells were obtained from American Type Culture Collection (Manassas, VA, USA). The cells were seeded at a density of 2 × 10^4^ cells/well in 96-well plates before being transfected with vectors (100 ng/well). Forty-eight hours after seeding, the medium was replaced and miScript miRNA mimic from Qiagen was introduced into the cells using Lipofectamine 3000 reagent. The miRNAs were used at a final concentration of 10 nM. Negative control cells were treated with small RNAs and scrambled sequences. After another 24 h, luciferase activity was measured with a luminometer from PerkinElmer (Waltham, MA, USA) and a Dual-Luciferase Reporter Assay System from Promega (Madison, WI, USA), following the manufacturer’s instructions, to determine the levels of miRNA expression.

### 2.11. Statistical Analyses

The statistical analyses were conducted using JMP version 14 from SAS Institute (Cary, NC, USA). The data are presented as the mean ± standard deviation (SD). To determine the significance of differences between the control and HFCS groups, either a parametric one-way analysis of variance with Tukey’s post hoc test or a nonparametric Wilcoxon test was utilized. Statistical significance was established as *p* < 0.05.

## 3. Results

### 3.1. Comparison of Body Weight and Caloric Intake of Offspring

The body weight and caloric intake in offspring were measured during PD21-60. No significant differences were observed between the offspring in the control and HFCS groups (Figure S1). These results are consistent with our previous study [10].

### 3.2. Comparison of Circulating GC Levels in Offspring

**Figure 1:**
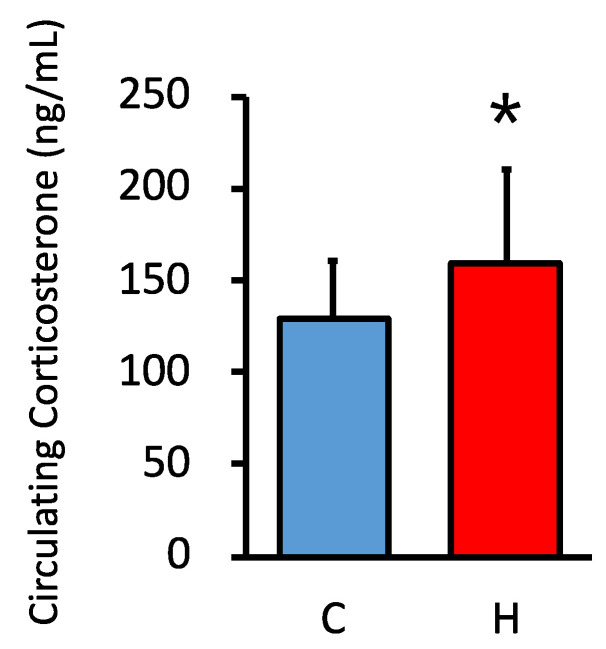
Maternal HFCS intake increased circulating corticosterone levels in offspring at PD60. C, control offspring (*n* = 12); H, offspring from HFCS-fed dams (*n* = 12). Values are presented as the mean ± SD. * *p* < 0.05. (HFCS; high-fructose corn syrup).

We investigated the effect of maternal HFCS intake on circulating GC levels in offspring. Circulating GC levels were significantly higher in the HFCS group than in the control group, as previously reported (Figure 1) [17].

### 3.3. Comparison of 11β-Hsd1 and 11β-Hsd2 Activities in the Liver, Kidney, Adrenal Glands, Muscle, and WAT in Offspring

**Figure 2:**
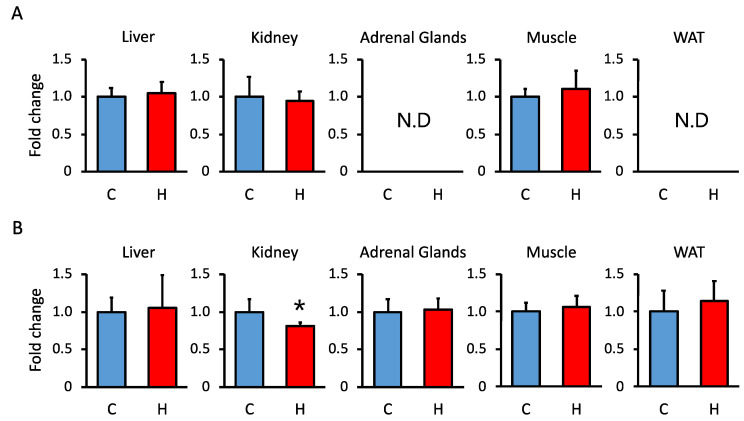
Effect of maternal HFCS intake on (**A**) 11β-Hsd1 and (**B**) 11β-Hsd2 activities in the liver, kidney, adrenal glands, muscle, and WAT of offspring at PD60. Metabolite levels were determined by HPLC. C, control offspring (*n* = 4–7); H, offspring from HFCS-fed dams (*n* = 3–5). Values are presented as the mean ± SD. * *p* < 0.05. (HFCS; high-fructose corn syrup, HPLC; high-performance liquid chromatography, N.D; not detected, WAT; white adipose tissue, 11β-Hsd; 11 beta-hydroxysteroid dehydrogenase).

To determine the mechanism by which maternal HFCS intake increased the circulating GC levels in offspring, we analyzed 11β-Hsd1 and 11β-Hsd2 activities in the liver, kidney, adrenal glands, muscle, and WAT. We observed a decrease in 11β-Hsd2 activity in the kidney and no significant changes in 11β-Hsd1 and 11β-Hsd2 in other tissues (Figure 2). To determine whether the decrease in renal 11β-Hsd2 activity was due to fructose or sugar, we investigated the effects of maternal glucose intake. There was no significant decrease in renal 11β-Hsd2 activity in the pups of mothers fed the 20% glucose solution (Figure S2). We also investigated the GC synthesis pathway, but found no significant changes in associated mRNA expression (Figure S3). These results suggest that reduced renal 11β-Hsd2 activity contributes to circulating GC levels in offspring of HFCS-fed dams.

### 3.4. Comparison of the mRNA and Protein Expression of 11β-Hsd2 in Offspring Kidney

**Figure 3:**
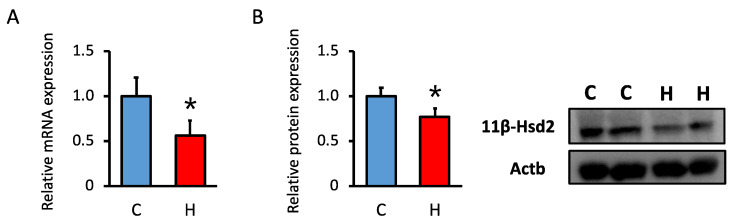
Effect of maternal HFCS intake on renal 11β-Hsd2 protein and mRNA levels. (**A**) The mRNA levels were quantified by qPCR. The gene expression levels are shown relative to *Actb.* (**B**) The protein levels were quantified by western blotting. The protein expression levels are shown relative to Actb. C, control offspring (*n* = 4–6); H, offspring from HFCS-fed dams (*n* = 4, 5). Values are presented as the mean ± SD. * *p* < 0.05. (Actb; β-actin, HFCS; high-fructose corn syrup, 11β-Hsd2; 11 beta-hydroxysteroid dehydrogenase 2).

Considering the decrease in renal 11β-Hsd2 activity following maternal HFCS intake, we investigated the mRNA and protein expression of 11β-Hsd2. The results showed attenuated mRNA (Figure 3A) and protein of 11β-Hsd2 expression (Figure 3B) in the kidney. Thus, the decrease in 11β-Hsd2 activity may be caused by the suppression of mRNA and protein levels.

### 3.5. Comparison of DNA Methylation Levels in Renal Hsd11b2 Promoter Region in Offspring

**Figure 4:**
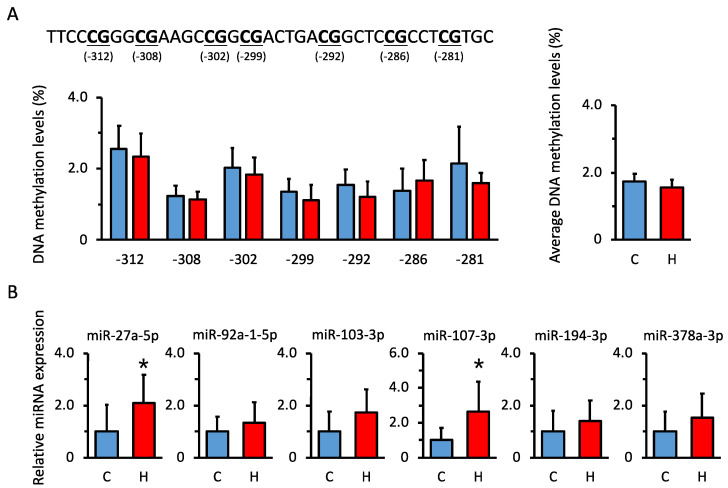
Epigenetic analysis of renal *Hsd11b2* mRNA expression. (**A**) DNA methylation levels of the *Hsd11b2* promoter region in the kidney. (**B**) Renal expression levels of miRNAs are predicted to target *Hsd11b2* mRNA and are highly expressed in the kidney. C, control offspring (*n* = 5–7); H, offspring from HFCS-fed dams (*n* = 4–6). Values are presented as the mean ± SD. * *p* < 0.05.

The transcription of *Hsd11b2* mRNA is regulated by DNA methylation [29,30] and miRNAs [31]. Thus, we determined whether maternal HFCS intake induces epigenetic changes. First, we used bisulfite sequencing to examine methylation levels in the *Hsd11b2* promoter region. No significant changes in the DNA methylation levels were observed in either group (Figure 4A).

### 3.6. Comparison of miRNA Expression Levels That Are Predicted to Be Biological Targets of Hsd11b2 mRNA in Offspring

Second, we analyzed the expression levels of miRNAs that are predicted to be biological targets of *Hsd11b2* mRNA and are highly expressed in the kidney. The miRNAs that were predicted to be the most potent (miR-27a-5p, miR-92a-1-5p, miR-103-3p, miR-107-3p, miR-194-3p, and miR-378-3p) were quantified by qPCR. The expression of miR-27a and miR-107 was significantly upregulated among these miRNAs (Figure 4B).

### 3.7. Analysis of the miRNA Suppression of Hsd11b2 mRNA

**Figure 5:**
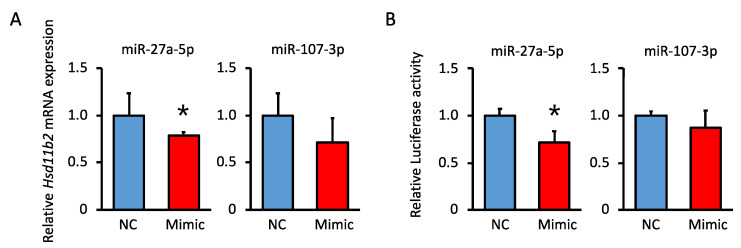
Regulation of *Hsd11b2* mRNA expression by miR-27a and miR-107 mimic. (**A**) The effects of miRNAs mimic *Hsd11b2* mRNA expression. The mRNA levels were quantified by qPCR. (**B**) Effects of miRNAs mimic luciferase activity. The luciferase activities were determined in cells transfected with miR-27a or miR-107 and luciferase vectors containing putative miRNA target sequences. Cells were also transfected with scrambled miRNA as a negative control. NC, Cells transfected with scrambled miRNA (*n* = 3, 4); Mimic, Cells transfected with miR-27a or miR-107 (*n* = 3, 4). Values are presented as the mean ± SD. * *p* < 0.05.

We determined whether miR-27a and miR-107 affected the expression of *Hsd11b2* mRNA in vitro. The miR-27a or miR-107 mimic was transfected into the C9 cells. *Hsd11b2* mRNA expression was reduced by transfection with miR-27a mimic in the C9 cells (Figure 5A). We performed a luciferase assay to investigate the effects of miRNAs on luciferase activity in HepG II cells. Luciferase activity was measured after transfection of cells with luciferase vectors containing sequences predicted to be targets of miRNAs, and transfection with miR-27a or miR-107. When the cells were transfected with the miR-27a mimic and its putative target sequence-containing vector, luciferase activity decreased (Figure 5B).

## 4. Discussion

In the present study, we observed decreased 11β-Hsd2 activity in the kidney of the HFCS group, which led to increased circulating GC levels. Epigenetic analysis revealed that miR-27a and miR-107 levels increased in the kidney. Transfection experiments showed that the transcription levels of *Hsd11b2* mRNA are regulated by miR-27a. Based on these results, the maternal HFCS consumption-induced elevation of circulating GC levels in offspring may be explained by a decrease in 11β-Hsd2 activity via renal miR-27a expression.

Maternal fructose consumption has been associated with DOHaD, which is characterized by insulin resistance, dyslipidemia, and other metabolic disorders in offspring [7,8,9,11,12,32]. However, the underlying mechanisms are poorly understood. Previous research has indicated that maternal fructose intake increases circulating GC levels in offspring [16,17], although the mechanisms underlying this phenomenon remain unclear. In the present study, we investigated the effects of maternal HFCS intake on offspring by measuring circulating GC levels and 11β-Hsd1 and 11β-Hsd2 activities in various tissues. The circulating GC levels were higher in the HFCS group than in the control group. Additionally, we observed a decrease in renal 11β-Hsd2 activity in the HFCS group, which could have contributed to increased GC levels. This decrease in renal 11β-Hsd2 activity may have been caused by the suppression of gene expression due to the upregulation of miR-27a and miR-107. These findings suggest that maternal HFCS intake induces miRNA-mediated epigenetic changes that may contribute to altered 11β-Hsd2 expression and circulating GC levels in offspring.

The results of the present study imply that the maternal intake of HFCS has notable long-term consequences for the metabolic health of offspring. Therefore, it is imperative to devise approaches to prevent or alleviate these effects. One potential method is to reduce the consumption of HFCS during pregnancy. This could be accomplished by imparting relevant findings to expectant mothers regarding the possible hazards of over-indulgence in fructose and by promoting healthier dietary patterns. Furthermore, the findings of this study suggest that targeting the miRNA-mediated regulation of *Hsd11b2* mRNA expression could be a potential therapeutic strategy for preventing or treating metabolic disorders associated with DOHaD. The suppression of miR-27a, miR-107, or other miRNAs implicated in the regulation of *Hsd11b2* mRNA expression could enhance the activity of this enzyme and restore the balance of GC levels in offspring. Additional research is required to investigate these interventions and their potential implementation in clinical practice.

It is widely acknowledged that the synthesis of GCs is regulated by adrenocorticotropic hormone (ACTH), which stimulates the expression of GC synthetic molecules [33]. When ACTH is released, adrenal GC synthesis is induced, leading to a greater than 10-fold increase in serum GC levels [34]. Moreover, research indicates that even slightly elevated GC levels can be a causative factor in various diseases [35,36]. For example, in a study of 2527 adults aged 54 to 87 years, cortisol levels in obese individuals were at most only 10% higher than in healthy individuals [35]. In addition, Kamba et al. reported an association between increased cortisol levels and decreased insulin secretion in 1071 Japanese adults [36]. In that study, there was a decrease in insulin secretion and an approximate 20–30% increase in the levels of serum GC above the normal baseline levels. Therefore, it is conceivable that an increase in the levels of GC, even a slight increase, may be sufficient to cause disease. In the present study, we observed an elevated baseline circulating GC levels (1.2-fold) with no transcriptional activation of GC synthetic molecules (Figure S3). Elevated GC levels in offspring may result from transgenerational toxicity caused by maternal fructose consumption.

We demonstrated renal 11β-Hsd2 activity in offspring, indicating that maternal fructose intake impairs renal function. This finding is consistent with the results from several studies that indicate the kidney is susceptible to fructose. Experiments on rats have revealed that a high-fructose diet causes tubulointerstitial inflammation and fibrosis [37]. Moreover, tubular cells are sensitive to fructose, and inflammation is even induced by endogenously produced fructose [38]. In addition to their high sensitivity to fructose, the kidney has a high capacity for fructose absorption. The kidney exclusively expresses one of the fructose transporters (Sglt5) and actively takes up fructose into cells. Björkman and Felig reported that when fructose is infused intravenously into humans, 20% of the infused fructose is taken up by the kidney [39]. Therefore, although the kidney is highly sensitive to fructose, they are also susceptible to fructose exposure. Consequently, it is possible that the kidney of the offspring is exposed to fructose via their dams, resulting in decreased renal function.

Maternal fructose may induce an increase in miR-27a expression in tubular cells, given that 11β-Hsd2 is expressed in the tubules [40]. Hanousková et al. previously showed that feeding mice high-fructose drinks increase miR-27a expression [41], which is consistent with the present results. Although the underlying mechanism is unclear, another study showed that miR-27a expression is upregulated by inflammation [42]. Given the link between maternal nutritional status and renal inflammation in offspring, maternal fructose consumption-induced inflammation may also increase miR-27a expression. This presents an interesting avenue for future research.

In the present study, 60-day-old offspring born to HFCS-fed dams exhibited decreased renal 11β-Hsd2 expression, whereas no decrease in expression was observed in the adrenal glands. In contrast, the authors of a previous study reported reduced 11β-Hsd2 expression in the adrenal glands of offspring at 160 days of age. These conflicting results can be attributed to the type of liquid used: HFCS or pure fructose. It has been suggested that there is a difference in the magnitude of the effects of pure fructose and HFCS [43,44]. Mock et al. showed that the decrease in *Ppara* mRNA expression induced by HFCS was greater than that induced by pure fructose [43]. Similarly, our previous DOHaD studies with HFCS or pure fructose showed an increase in GC at 60 days of age, but not with pure fructose (observed at 160 days of age). Therefore, the timing of the appearance of offspring phenotype may differ depending on whether the dam ingests HFCS or pure fructose.

The present study has several limitations. First, the epigenetic analysis was only performed at 60 days of age. Longitudinal analysis is required to determine when miRNA expression changes. Second, only male offspring were included in the study. We need to determine whether the same phenomenon is observed in females. Third, only enzyme activity analysis was performed for 11β-Hsd1. Future longitudinal analysis may reveal changes in 11β-Hsd1 enzyme activity and gene expression.

## 5. Conclusions

In summary, we suggest that maternal HFCS intake decreases renal 11β-Hsd2 activity and increases miR-27a expression in the kidney, resulting in increased GC levels. The present study allowed us to determine one of the mechanisms underlying GC elevation in offspring, which is often observed in DOHaD.

## Data Availability

Data are contained within the article.

## Supplementary Materials

The following supporting information can be downloaded at: https://www.mdpi.com/article/10.3390/nu15092122/s1, Figure S1: No effect on body weight during each period of offspring, Figure S2: Effect of maternal glucose intake on 11β-Hsd2 activity in the kidney on PD60, Figure S3: Effect of maternal HFCS intake on mRNA expression in the adrenal GC synthesis pathway.

## Abbreviations

DOHaD	developmental origins of health and disease
GC	glucocorticoid
HFCS	high-fructose corn syrup
miRNA	microRNA
WAT	white adipose tissue
11β-Hsd1	11 beta-hydroxysteroid dehydrogenases 1
11β-Hsd2	11 beta-hydroxysteroid dehydrogenases 2
