# Supplementary material for: Maternal High-Fructose Corn Syrup Intake Impairs Corticosterone Clearance by Reducing Renal 11β-Hsd2 Activity via miR-27a-Mediated Mechanism in Rat Offspring
Source: Nutrients. 2023 Apr 28;15(9):2122. doi: 10.3390/nu15092122 (PMC10180772; doi:10.3390/nu15092122)
Supplement: Supplementary file 1 [file nutrients-15-02122-s001.zip › nutrients-2347538-SI.pdf]

Supplementary Materials

Maternal high-fructose corn syrup intake impairs corticosterone clearance by reducing renal 11β-Hsd2 activity via miR-27a-mediated mechanism in rat offspring

Supplementary Figures

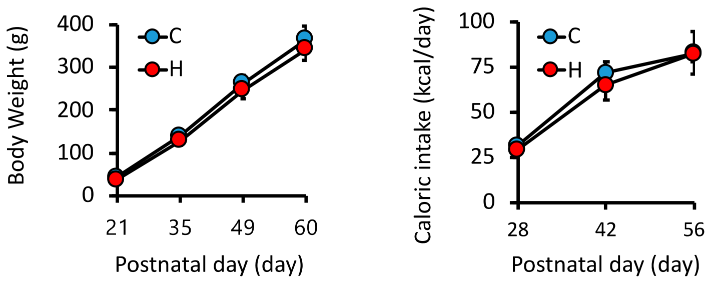

Figure S1. No effect on body weight during each period of offspring. Changes in body weight of rats every 14 days. There were no significant differences between groups C and H. C: control offspring (n = 14); H: offspring from HFCS-fed dams (n = 12). Values are presented as the mean ± SD. (HFCS; high-fructose corn syrup).

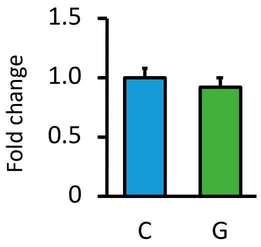

Figure S2. Effect of maternal glucose intake on 11β-Hsd2 activity in the kidney on PD60. 11β-Hsd2 activity was calculated based on the conversion of cortisol to cortisone. The levels of the metabolites were determined by HPLC. C, control offspring (n = 6); G, offspring from 20% glucose solution-fed dams (n = 6). (HPLC; high-performance liquid chromatography, 11β-Hsd2; 11 beta-hydroxysteroid dehydrogenase 2).

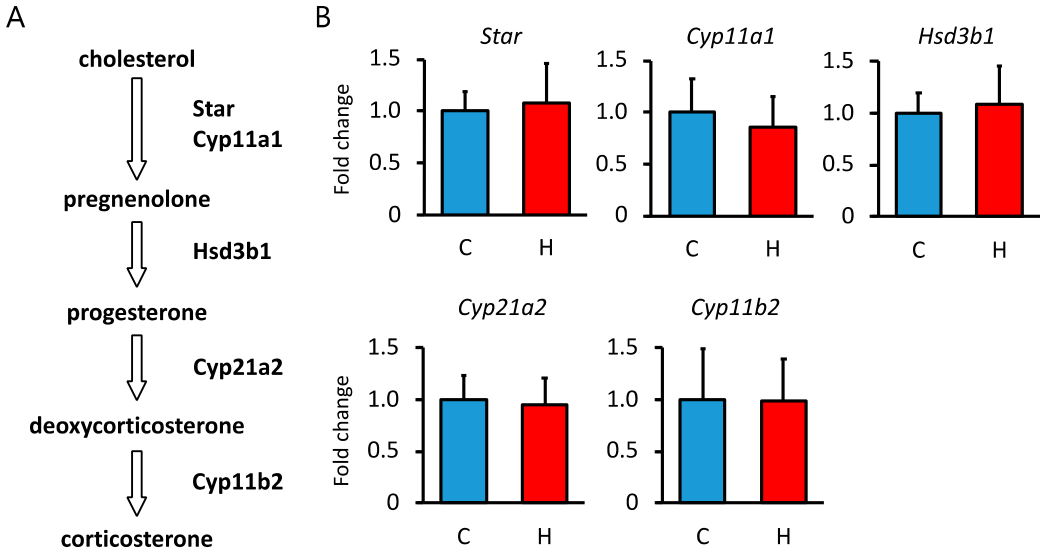

Figure S3. Effect of maternal HFCS intake on the adrenal GC synthesis pathway. (**A**) The scheme of adrenal GC synthesis pathway and GC synthesis enzymes. (**B**) The mRNA expression of the adrenal GC synthesis enzymes was quantified by qPCR. The gene expression levels are shown relative to *Actb*. C, control offspring (n = 6, 7); H, offspring from HFCS-fed dams (n = 6, 7). Values are presented as the mean ± SD. (GC; glucocorticoid, HFCS; high-fructose corn syrup, Star; steroidogenic acute regulatory protein, Cyp11a1; cytochrome monooxygenase, Hsd3b1; 3β-hydroxysteroid dehydrogenase, Cyp21a2; steroid 21-hydroxylase, Cyp11b2; aldosterone synthase).
